# Structuring a conceptual model for cost-effectiveness analysis of frailty interventions

**DOI:** 10.1371/journal.pone.0222049

**Published:** 2019-09-11

**Authors:** Hossein Haji Ali Afzali, Jonathan Karnon, Olga Theou, Justin Beilby, Matteo Cesari, Renuka Visvanathan

**Affiliations:** 1 College of Medicine and Public Health, Flinders University, Adelaide, South Australia, Australia; 2 Department of Medicine, Dalhousie University, Halifax, Nova Scotia, Canada; 3 Torrens University, Adelaide, South Australia, Australia; 4 Department of Clinical Sciences and Community Health, University of Milan, Milan, Italy; 5 Adelaide Medical School, The University of Adelaide, Adelaide, South Australia, Australia; Icahn School of Medicine at Mount Sinai, UNITED STATES

## Abstract

**Background:**

Frailty is a major health issue which impacts the life of older people, posing a significant challenge to the health system. One of the key emerging areas is the development of frailty interventions to halt or reverse the progression of the condition. In many countries, economic evidence is required to inform public funding decisions for such interventions, and cost-effectiveness models are needed to estimate long-term costs and effects. Such models should capture current clinical understanding of frailty, its progression and its health consequences. The objective of this paper is to present a conceptual model of frailty that can be used to inform the development of a cost-effectiveness model to evaluate frailty interventions.

**Methods:**

After critical analysis of the clinical and economic literature, a Delphi study consisting of experts from the disciplines of clinical medicine and epidemiology was undertaken to inform the key components of the conceptual model. We also identified relevant databases that can be used to populate and validate the model.

**Results:**

A list of significant health states/events for which frailty is a strong independent risk factor was identified (e.g., hip fracture, hospital admission, delirium, death). We also identified a list of important patient attributes that may influence disease progression (e.g., age, gender, previous hospital admissions, depression). A number of large-scale relevant databases were also identified to populate and validate the cost-effectiveness model. Face validity of model structure was confirmed by experts.

**Discussion and conclusions:**

The proposed conceptual model is being used as a basis for developing a new cost-effectiveness model to estimate lifetime costs and outcomes associated with a range of frailty interventions. Using an appropriate model structure, which more accurately reflects the natural history of frailty, will improve model transparency and accuracy. This will ultimately lead to better informed public funding decisions around interventions to manage frailty.

## Introduction

Frailty is a state of increased vulnerability to stressors, arising from cumulative impairments in multiple body systems characterised by significant decreased homeostatic reserves [1, 2]. Linked with population ageing, it is associated with increased risk of important events such as hip fracture, disability, and hospital admissions with consequential impact on quality of life and health care expenditures [3–5]. Globally, it has been estimated that between 4% and 17% of older people are frail [6]. Frailty status is commonly measured using two main approaches: the Frailty Phenotype (FP; representing a physical frailty definition) and the Frailty Index (FI; representing a deficit accumulation model incorporating multiple domains). The FP approach consists of five variables (i.e., unintentional weight loss, self-reported exhaustion, weak grip strength, slow walking speed, and sedentary behaviour), and classifies frailty into frail (individuals with three or more factors) and pre-frail (individuals with one or two factors). Pre-frail individuals are at increased risk of progression to frailty and, compared with frail people, have an intermediate risk of adverse health outcomes [7]. In the FI approach, frailty is measured as the proportion of deficits present in an individual (for example, an individual with 20 deficits from a total of 50 deficits measured, would have an FI score of 0.4) [8].

With no effective interventions, frailty is a progressive condition typically transitioning from lesser to greater states of the condition [9]. This is an important issue given the projected growth in the number of people living with frailty [10]. Emerging evidence suggest that timely interventions can delay frailty progression or increase the likelihood of transitioning to less severe states [11]. One of the key emerging area is the development and evaluation of frailty interventions to reduce frailty or delay the progression of frailty. In many countries such as Australia and the UK, economic evaluation is a key part of public funding decisions for new interventions and is used to demonstrate that the additional effects of a new intervention justify its additional cost. In most cases, the preferred unit of health improvement is the quality-adjusted life-year (QALY) which is a composite measure of both mortality and quality of life. One QALY represents one year of life in full health.

A few within-trial economic evaluations have assessed the cost-effectiveness of frailty interventions. Fairhall et al. [12] undertook an economic evaluation alongside a randomised controlled trial to evaluate the costs and effects of a physiotherapy-based intervention. They found that the intervention was more costly than usual care (mean difference: AUD 2,145) with no significant difference in QALYs over the 12-month follow-up. A recent within-trial economic evaluation of nurse-led multifactorial care found that at 12 months the mean costs were significantly higher in the intervention group compared with usual care with no significant differences in QALYs [13]. Another study assessed a 1-year case management intervention in comparison to usual care for frail people and reported no differences in costs and QALYs between the two groups [14].

The results of the above economic evaluations indicate that the proposed new interventions are not deemed to be cost-effective. However, the costs and health outcomes in these studies were reported over a short time horizon. Given the natural history of frailty, it is likely that some important health outcomes and costs may not have been captured within the trial time horizons (e.g., QALY gains beyond 12 months as a result of transition to lesser states of frailty or averted costs due to reduced hospital admissions). One method to address this limitation is the use of cost-effectiveness models. Such models predict the experience of relevant health states related to the condition under study, often over the remaining lifetime of a study population. Costs and quality of life weights are applied to the time spent in different health states to estimate the effects of health care interventions on longer-term (e.g., lifetime) costs and outcomes.

Karnon et al. [15] developed a cost-effectiveness model to predict lifetime costs and QALYs of pre-frail and frail individuals evaluating the physiotherapy-based intervention assessed by Fairhall et al. [12]. Unlike the within-trial analysis, this paper concluded that the intervention, compared to usual care, could be cost-effective. This finding demonstrates the importance of the use of models in extrapolating costs and outcomes beyond the follow-up period of clinical trials.

It is well accepted that the accuracy of estimates of costs and outcomes obtained from cost-effectiveness models are driven by the model development process, including the specification of model structure (e.g., health states/events included in the model) [16]. In the literature, the impact of inappropriate choice of model structure on model outputs (and hence funding decisions) has been well noted [17–23]. The structure of the model developed by Karnon et al. [15] included some important health states with evidence of strong associations with frailty (e.g., depression, fractures, and residential care admissions). However, the model structure was driven mainly by data availability rather than through a systematic model conceptualisation process. Recent guidelines for good modelling practice highlight the need for the development of a conceptual model as a basis for defining the structure of cost-effectiveness models [24, 25]. A conceptual model should reflect the current clinical understanding of the condition under study, not the availability of data to populate a model.

The objective of this paper is to present a conceptual model of the progression and consequences of frailty that can be used to guide the development of a cost-effectiveness model (to be reported separately) for the economic evaluation of frailty interventions. In the health economic modelling literature, a very limited number of studies reported the model conceptualization process, for example, Ramos et al. [16] and Tabberer et al. [26] who developed conceptual models of primary asthma prevention in children and Chronic Obstructive Pulmonary Disease (COPD) respectively. As noted by Ramos et al. [16] reporting on the model development process will increase transparency and credibility of cost-effectiveness models, similar to the process of reporting study protocols separate from the clinical trial results. We also identify a number of relevant databases which can be used to populate and validate the model.

## Methods

To develop a frailty conceptual model, we used published guidance on good modelling practice including the recommendations made by joint Society for Medical Decision Making (SMDM)/ International Society for Pharmacoeconomics and Outcomes Research (ISPOR) Modelling Task Force [24] and the National Institute for Health and Care Excellence (NICE) Technical Support Document (TSD) on model conceptualisation [27].

The model conceptualisation process is represented by two key choices. First, represented health states/events should be clinically relevant and ‘significant’. ‘Significant’ health states/events are defined with respect to the strength of relationship between the condition of interest (e.g., frailty) and the health state/event, as well as their potential impact on associated costs and/or meaningful health outcomes (life expectancy/quality of life). Another key structural choice concerns ‘important’ patient attributes that may influence frailty progression. The attributes may include socio-demographic characteristics such as age and gender, prior clinical events (e.g., previous fractures increase risk of fractures in frail people) and measures of health status (e.g., ability to undertake activities of daily living).

### Literature review

To inform the conceptual model, first a comprehensive clinical literature review was conducted to study current knowledge of frailty and to develop initial clinical insight into the natural course of frailty. We identified studies (including clinical practice guidelines) documenting disease progression that reported important health states/events associated with frailty and relevant patient attributes. The literature search of PubMed was conducted during May 2017. The search was limited to articles published in English from 2000 onwards (See S1 Appendix for details). The health economics literature was also reviewed to identify any existing frailty cost-effectiveness models. This can help identify the structural choices made in previous models, and inform the significance of model structural aspects from the economic perspective.

### Expert engagement

To further guide the development of the conceptual model, a group of experts was selected. Expert engagement is a recognised tool in informing key structural choices within the conceptualisation process in the field of decision analytic modelling [28, 29]. Two advisory groups were convened to help develop the conceptual model. The first was the project Management Group consisting of experienced frailty clinicians/researchers (with expertise in areas such as geriatrics, nursing, orthopedic surgery, rehabilitation medicine and epidemiology) and health economists (with extensive experience in cost-effectiveness modelling). The group received the report of the findings of the literature review. Two sessions were planned during which the group was asked to review and refine the draft and to guide the development of an online survey for the second group. The group was also asked to pilot test the survey.

The second advisory group was convened as a Delphi panel to collate the opinions of experts and reach consensus over the structural choices included in the conceptual model. Delphi methods have been previously used for gaining expert consensus in social sciences [30–32]. In Delphi studies, the selection of participants is one of the most important steps as it directly impacts the quality of the data generated [33]. In consultation with the Management Group, twelve Australian and international frailty experts were invited. After providing information on the study including its aim, design and potential outputs, the experts were asked if they are willing to participate in the study. Nine agreed to participate including six geriatricians, two epidemiologists (with expertise in epidemiological and clinical studies of frailty and aging) and one general practitioner (with experience in frailty screening and management in primary care). After obtaining written consents (via emails), three rounds of online survey were conducted by emails (between August and December 2017). In each round, one follow-up email was sent if participants did not respond within two weeks (See S2 Appendix for the list of questions).

In round 1, the experts were asked to review the health state/events associated with frailty and patient attributes identified during previous steps. In this round, the participants were asked to 1) estimate the strength of the relationships between frailty and the identified health states/events on a scale of 1–5 (1 = ‘Very weak’; 5 = ‘Very strong’); 2) qualitatively specify the magnitude of influence of the patient attributes on disease progression on a scale of 1–5 (1 = ‘Very weakly’; 5 = ‘Very strongly’) 3) suggest additional important health states/events and patient attributes that might be missing from our list. In round 2, the participants were asked to assess the significance of the health states/events with respect to their potential impact on mortality/quality of life/resource use on a 5-point scale (from 'very low impact' to 'very significant impact').

After analysing the findings of rounds 1 and 2, we classified the structural choices into three pre-specified categories based on the level of agreement among the participants on their relevance and significance, as used by previous Delphi studies [34, 35]:

- Acceptable level of agreement (and hence included in the proposed conceptual model):
  - Health states/events: a mean score of 4 to 5 with respect to strength of a health state/event’s relationship with frailty (‘strong’ to ‘very strong’ association) and impact on mortality/quality of life/resource use (‘significant’ to ‘very significant’ impact); as well as at least 80% of the participants scored a 4 or 5.
  - Patient attribute: a mean score of 4 to 5 (‘strong’ to ‘very strong’ influence on frailty progression) with at least 80% of the participants scored a 4 or 5.
- Moderate level of agreement:
  - Health states/events: a mean score of 3 to 4 with respect to strength of a health state/event’s relationship with frailty (‘intermediate’ to ‘strong’ association) and impact on mortality/quality of life/resource use (‘medium’ to ‘significant’ impact); as well as with at least 60% of the participants scored at least a 4. Health states/events with a moderate level of agreement with respect to both aspects (or with a moderate level of agreement with respect to one aspect and an acceptable level of agreement with regard to the other aspect) were fed back to participants (see round 3 below).
  - Patient attributes: a mean score of 3 to 4 (‘intermediate’ to ‘strong’ influence on frailty progression) with at least 60% of the participants scored at least a 4. These attributes were fed back to the participants.
- Unacceptable level of agreement: All structural choices with other than acceptable or moderate level of agreement were rejected.

Round 3 involved a personalised survey. In this round, we fed back all health states/events and patient attributes with a moderate level of agreement, along with the individual participant’s previous scores. This provided opportunities for the participants to modify their responses from previous rounds. If an acceptable level of agreement was found, we would include the structural choices in the proposed conceptual model.

## Results

The review of the clinical literature found 28 health states/events for which frailty is considered an independent risk factor. Some examples include falls, hip fracture, vertebral fracture, hospital admissions, residential care admission, hearing and vision impairments, cognitive impairments, depression, polypharmacy, physical disability, obesity, heart failure, stroke and death (See S1 Table: List of health states/events). The review of the health economics literature found that Karnon et al. [15] is the only published model-based economic evaluation of frailty interventions. As discussed before, the model structure included admission to residential care, fracture, depression and death as health states/events with frailty status and number of previous fractures as patient attributes.

All nine experts participated in three rounds of the Delphi study. In the first and second rounds, experts reviewed the findings of the literature review and an acceptable level of agreement was found with respect to six health states/events including hip fracture, falls, residential care admission, hospital admission, physical disability and death. A moderate level of agreement was found with respect to five health states/events including other fractures (all fractures excluding hip and vertebral fractures), delirium, incontinence, polypharmacy and cognitive impairments. These health states/events were provided to the participants in the final round of the Delphi study, and an acceptable level of agreement was found with respect to delirium.

The literature review identified 27 patient attributes which may influence the progression of frailty. Some examples include age, gender, polypharmacy, stroke, diabetes, depression, arthritis, smoking status (See S2 Table: List of patient attributes). A bidirectional relationship between frailty and some conditions was found. For example, polypharmacy and depression were identified as risk factors as well as health states/events associated with frailty. The Delphi process identified 10 patient attributes with an acceptable level of agreement including age, gender, level of education, frailty status, number of previous hospital admissions, number of previous fractures, polypharmacy, stroke, diabetes and level of physical activity. These attributes were included in the conceptual model. A moderate level of agreement was found with respect to four attributes including smoking status, living area, depression and cognitive impairments. In round 3, only depression was identified as an attribute with an acceptable level of agreement. The findings of the Delphi study informed the revised conceptual model which was sent to all participants for their final review and approval.

### Modelling technique

Another key structural choice is around the type of modelling technique (i.e., implementation framework), which should be informed by the findings of the conceptualisation process [21]. [36, 37]. By allowing for evaluation of recurring events, cohort-based state-transition (Markov) models are commonly used in modelling chronic conditions. However, one of the key limitations associated with Markov models is the Markovian assumption, which relates to the lack of memory, i.e., transition probabilities are not influenced by patient attributes and previous health states. This can be dealt with by disaggregating health states to model subgroup characteristics. However, the process can soon lead to an unwieldy number of health states [36, 37].

Based on understanding of frailty progression, patients’ characteristics (e.g., age, frailty status) and history of events (e.g., fracture) play an important role in experiencing future events. For example, the risk of a subsequent fracture is influenced by frailty status and a prior fracture. Among patient attributes, frailty status (commonly measured by the FI or FP approach) is one of the key predictors of disease progression [38, 39]. Measures of frailty status are commonly used as primary outcomes in clinical studies and should be explicitly modelled [40–42].

**Fig 1:**
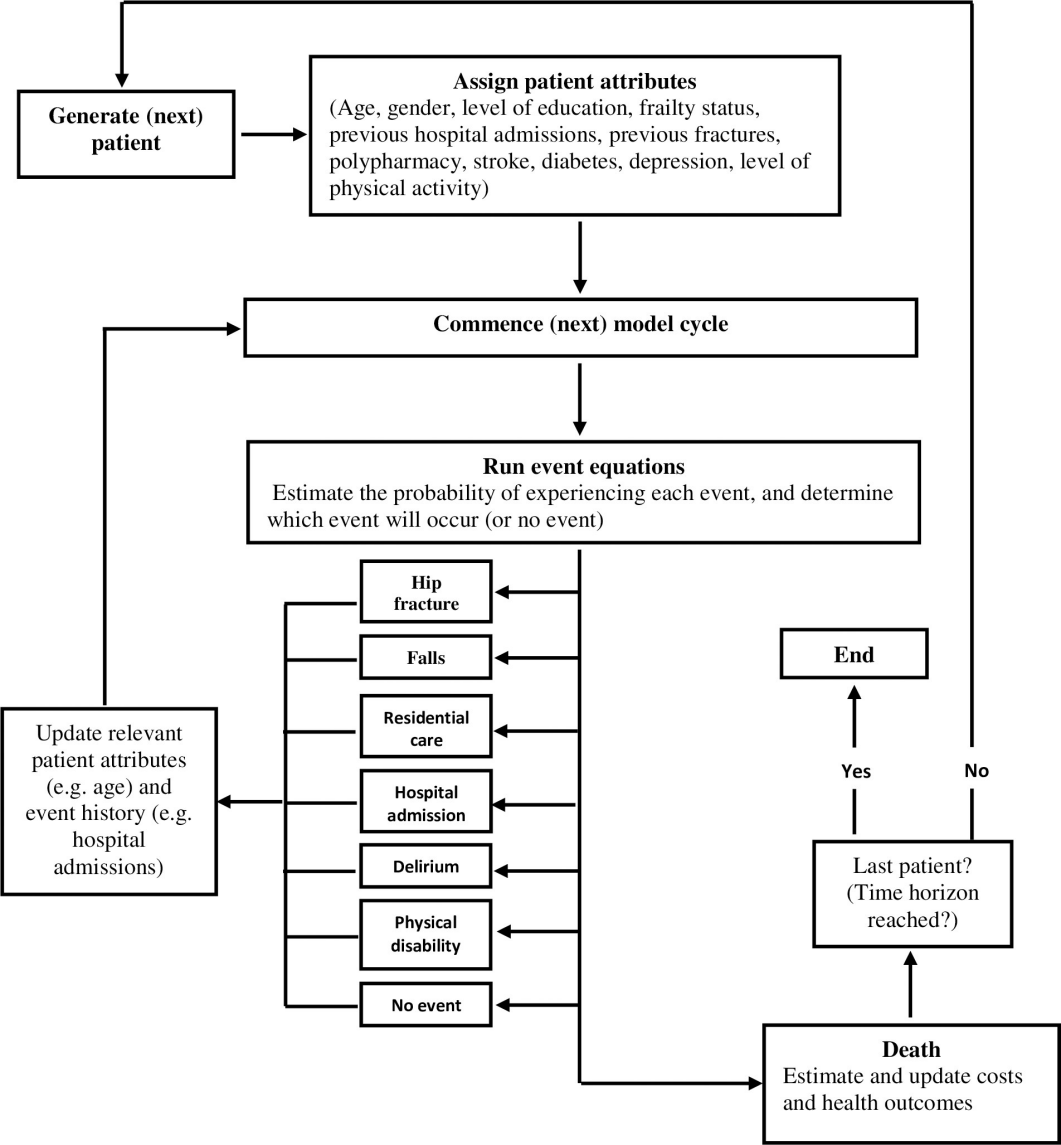
Flow of patients in the model.

Due to its flexibility to incorporate the memory of patient attributes and history of events to predict health outcomes, the most appropriate technique to model frailty progressions is an individual-based model. Fig 1 shows the schematic representation of the flow of patients in the model

As the figure shows, at the beginning of the model a patient is assigned relevant attributes (such as age, gender, and frailty status) and the simulation starts. After assigning relevant patient attributes, the risk of experiencing health events included in the model (e.g., fracture) is derived from risk equations defined as functions of, for example, age, gender and frailty status. For the next cycle, the model will update patient attributes (e.g., the attribute ‘number of previous fractures’ is increased by one) and use this event history to adjust the risk of having a subsequent fracture. As simulated patients experience health events, the model estimates QALYs and costs to quantify health outcomes and healthcare resources use. If the model predicts that the patient dies, the number of QALYs and costs are summed across the health events.

#### Potential data sources to inform the proposed model

The usefulness of the proposed structural choices will in part be determined by the availability of relevant data to inform model inputs. After reviewing existing databases, and in consultation with our advisory groups, a number of relevant databases were identified. Analyses of these databases, supplemented with data from published literature and administrative databases, provide sufficient data to inform model inputs including risk equations, utility values (to estimate QALYs) and costs associated with health states/events. The databases can also be used to validate the model [43]. This will involve comparisons between the model predictions and (i) observed data from databases used to populate the model (internal validity) (ii) observed data from databases that have not been used in the model construction (external validity).

The Survey of Health, Ageing and Retirement in Europe (SHARE) is a large cross-national panel database including data on health, socio-economic status and health care utilizations (e.g., hospital admissions) [44]. It comprises data from approximately 123,000 individuals aged 50 or older covering 27 European countries. To date, SHARE has collected and released data covering six panel waves (2004–2015). With the exception of delirium and advanced diabetes, the SHARE includes data on the health states/events and patient attributes included in the conceptual model. With over 50,000 observations, the Dynamic Analyses to Optimize Ageing (DYNOPTA) comprises harmonised data from nine Australian Longitudinal Studies of Ageing [45]. The database contains a range of data required to populate the model including socio-economic characteristics, frailty status, physical disability, depression, stroke, depression, physical activities, hospital admissions and mortality. The Healthy Ageing Research Consortium in Australia is generating the Registry of Older South Australian (ROSA) database [46]. Derived from the national aged care data, ROSA is a longitudinal dataset comprising data from approximately 2.9m individuals (>65yo) (1997–2014). Some examples of the ROSA data that can be used to populate the model include socio-demographic characteristics (e.g., age, gender, level of education), frailty status, falls, delirium, depression, diabetes, hip fracture, hospital admissions and residential care admissions.

## Discussion

The development of high-quality cost-effectiveness models is required to estimate costs and benefits beyond trial time horizons to capture all important differences in costs and outcomes. This paper has reported on the steps in developing a conceptual model of frailty as an underlying structure for a cost-effectiveness model. The cost-effectiveness model can be used to predict lifetime costs and effects of a range of frailty interventions that are designed to reduce the risk of adverse health outcomes (e.g. fracture, hospital admissions) by reversing frailty (i.e., increasing the likelihood of transitioning to lesser states of frailty), preventing frailty or delaying its progression. These interventions include various components focusing on, for example, nutritional deficiency, physical parameters of frailty (e.g., reduced balance), and cognitive function [47]. Clinical studies evaluating the effectiveness of frailty interventions commonly use change in patient’s frailty level (or score) as a primary outcome measure [11, 40]. Examples include a physiotherapy-based intervention (using the FP approach to measure frailty) [12] and home visits by health professionals (using the FI approach) [48], with usual care as a relevant comparator. A recent systematic review evaluating the effectiveness of frailty interventions found that the FP was the most common primary outcome of interest in clinical studies [40].

By explicitly modelling the level of frailty status (and other relevant patient characteristics), the cost-effectiveness model can translate the effects of frailty interventions (on changing frailty level) into improvements in economically meaningful health outcomes (e.g., QALYs, life expectancy) and reductions in health care costs which may offset some of the costs associated with interventions. Using SHARE, frailty status can be measured (and incorporated into the model) using both FP and FI approaches. The model will be flexible to take different perspectives (e.g., government, societal) depending on the aim of the evaluation and data availability (e.g., to estimate indirect costs).

The proposed conceptual model represents a number of significant health states/events and patient attributes, and was informed by a two-step approach; a comprehensive literature review followed by a Delphi process to achieve consensus among a group of frailty experts. The literature review identified a number of important and relevant health states/events and patient attributes. The experts contributed significantly to the revision of the conceptual model. The proposed conceptual model will be used to guide the development of a cost-effectiveness model ensuring that all key structural choices included in the model will be clinically and economically relevant and important. We also proposed an individual-based model as an appropriate modelling technique to implement the conceptual model.

One of the limitations when moving from the conceptual model to a cost-effectiveness model is the availability of quality data to inform all model inputs and validate the model. In the absence of relevant data, the conceptual model should be revised and simplified and the potential impact of simplifying assumptions on model outputs fully described. This was acknowledged in studies of conceptual models of asthma and COPD [16, 26]. For example, to populate the COPD conceptual model, the only relevant observational study identified by the authors did not include all relevant data, and hence some patient attributes (e.g., ethnicity) were excluded from the final proposed conceptual model. The authors did not discuss the potential impact of this omission on model outputs. We have identified a number of relevant data sources (e.g., SHARE, DYNOPTA, ROSA). Supplemented with published literature, these databases provide sufficient data to estimate all key inputs into the cost-effectiveness model.

Similar to other Delphi studies, one point of concern is that the model structure is highly dependent on the inputs from experts. The Delphi technique was used to inform conceptual cost-effectiveness models in the two previous studies of Asthma and COPD and its value has been widely discussed [16, 26]. We tried to improve the validity of expert inputs by recruiting a multi-disciplinary panel (i.e., clinicians, epidemiologist and health economists) with an in-depth knowledge of frailty who are often cited. Clinical experts play a key role in modelling disease progression [24]. Nevertheless, we acknowledge that the involvement of other stakeholders (such as community nurses, patients and caregivers) can further enhance the conceptualisation process.

Model validity is another key issue including face validity, internal validity, external validity and cross model validity [43, 49]. Face validity refers to subjective judgement on whether the structure of the model, data sources, and model predictions are sensible. Face validity of our model structure was confirmed by experts, similar to the studies reporting the development of conceptual models of Asthma and COPD [16, 26]. Experts also assessed the appropriateness of the data sources identified in our study to populate the model. After developing a frailty cost-effectiveness model, the assembled experts will also be asked to assess to the face validity of model predictions. Internal and external validity (as discussed before) will be assessed by comparing the model predictions and the observed data from the data sources identified. Cross-model validity will be assessed by comparing our model predictions and the estimates of frailty model developed by Karnon et al. [15] (the only published frailty model) to assess the potential impact of alternative model structures on model predictions.

It is well noted that the accuracy of model outputs are strongly influenced by the choices around key structural aspects [17–23]. Although recent modelling guidelines recommend a transparent, evidence-based process to develop a conceptual model as a basis to inform model structure, the model conceptualisation process has not been widely used in the modelling literature. Limited examples in the literature include Ramos et al. [16] who provided a detailed description of steps in structuring a cost-effectiveness model of primary asthma prevention in children, and Tabberer et al. [26] who described the process to develop a conceptual model of disease progression to assess the cost-effectiveness of the interventions for the management of COPD.

## Conclusions

This study reports on the development of a conceptual cost-effectiveness model of the progression of frailty to evaluate frailty interventions. A frailty cost-effectiveness model will estimate the costs and outcomes associated with frailty interventions over a lifetime horizon; one of the key inputs to inform public funding decisions. Such longer term costs and effects are often not captured in clinical trials due to the short observation periods. It is expected that explicitly detailing the steps used in the development of the conceptual model enhances communication between all stakeholders in model-based evaluations including the modeller, clinical experts and decision makers. It also improves model transparency, accuracy, and comparability and ultimately improves the efficiency of public funding decisions.

## Supporting information

S1 Appendix Search strategy. (PDF)

S2 Appendix List of questions. (PDF)

S1 Table List of health states/events. (PDF)

S2 Table List of patient attributes. (PDF)
