# Supplementary material for: Structuring a conceptual model for cost-effectiveness analysis of frailty interventions
Source: PLoS One. 2019 Sep 11;14(9):e0222049. doi: 10.1371/journal.pone.0222049 (PMC6738928; doi:10.1371/journal.pone.0222049)
Supplement: S1 Appendix — (PDF) [file pone.0222049.s001.pdf]

S1 Appendix

To inform the conceptual model, first a comprehensive clinical literature review was conducted to study current knowledge of frailty and to develop initial clinical insight into the natural course of frailty. We identified studies (including clinical practice guidelines) documenting disease progression that reported important health states/events associated with frailty and relevant patient attributes. The literature search of PubMed was conducted during May 2017. The search was limited to articles published in English from 2000 onwards Reference lists of key articles were cross-referenced by hand searching to identify additional articles. Additional references were also provided by the research team.

Search strategy

1	Geriatric Assessment/ or Aging/ or Frail Elderly/ or Frailty/ or Health Services for the Aged/
2	Pre-frail.mp
3	Prefrail.mp
4	Elder.mp
5	(Healthy aging or healthy ageing or successful aging or successful ageing).mp
6	exp Aged/
7	Or/1-6
8	Disabil*.mp or Disability Evaluation/
9	"Activities of Daily Living"/
10	functional decline.mp
11	Geriatric Assessment/
12	Patient Outcome Assessment/ or "Outcome Assessment (Health Care)"/ or "Outcome and Process Assessment (Health Care)"/ or Patient Reported Outcome Measures/(and patient attributed (MeSH))
13	Or/8-12
14	7 and 13
15	limit 14 to yr="2000 -2017"
16	limit 15 to “English” language

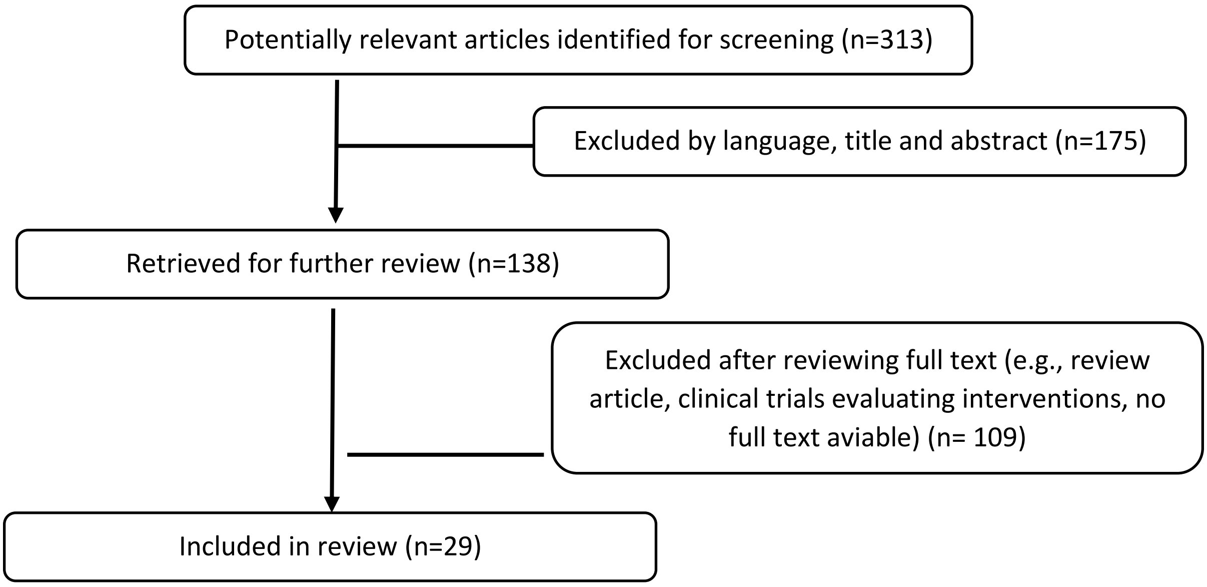

Flow diagram of included and excluded articles
