# Supplementary material for: Structuring a conceptual model for cost-effectiveness analysis of frailty interventions
Source: PLoS One. 2019 Sep 11;14(9):e0222049. doi: 10.1371/journal.pone.0222049 (PMC6738928; doi:10.1371/journal.pone.0222049)
Supplement: S2 Appendix — (PDF) [file pone.0222049.s002.pdf]

Thank you for participating in our survey. We greatly appreciate you taking the time to complete it.

This survey aims to identify important structural aspects of a frailty model.

The survey includes 2 sections and is expected to only take 10 minutes of your time. In the first section we focus on health outcomes. We present a number of "clinical states/events" for which frailty could be an independent risk factor (i.e. after controlling for other differences between frail and non-frail persons). We ask you to specify the extent to which frailty increases the risk of each state/event (magnitude of association). In the second section we need your inputs to specify a number of important "patient attributes" that influence the progression of frailty and the health outcomes identified in Section 1. We also ask you to identify additional important clinical states/events and patient attributes that have been missing from our list.

Please note that there are some conditions (e.g. depression) that have been presented in both sections. Our literature review has found a complex directional relationship between frailty and these conditions. While in some published studies frailty has been considered as an independent risk factor for these conditions, other studies have considered the conditions as an attribute that influences the progression of frailty and health outcomes.

What is your name?

What is your speciality?

SECTION 1: Health Outcomes (Specification and rating)

1.1 To what extent is frailty an independent risk factor for the following conditions?

	Frailty is **NOT** a risk factor	**Very weak** risk factor	**Weak** risk factor	**Intermediate** risk factor	**Strong** risk factor	**Very strong** risk factor
Hip fracture	○	○	○	○	○	○
Vertebral fracture	○	○	○	○	○	○
Other fractures (other than hip and vertebral)	○	○	○	○	○	○
Falls	○	○	○	○	○	○
Delirium	○	○	○	○	○	○
Incontinence	○	○	○	○	○	○
Pressure ulcers	○	○	○	○	○	○
Sleep disorders	○	○	○	○	○	○
Admission to a residential care facility	○	○	○	○	○	○
Hospital admissions	○	○	○	○	○	○
Polypharmacy	○	○	○	○	○	○
Heart failure	○	○	○	○	○	○
Angina	○	○	○	○	○	○
Myocardial infarction	○	○	○	○	○	○
Stroke	○	○	○	○	○	○
Diabetes	○	○	○	○	○	○
Obesity	○	○	○	○	○	○
Parkinson's Disease	○	○	○	○	○	○
Impaired vision	○	○	○	○	○	○
Impaired hearing	○	○	○	○	○	○
Chronic Obstructive Pulmonary Disease	○	○	○	○	○	○
Poor oral health	○	○	○	○	○	○
Peripheral artery disease	○	○	○	○	○	○
Renal failure	○	○	○	○	○	○
Depression	○	○	○	○	○	○

	Frailty is **NOT** a risk factor	**Very weak** risk factor	**Weak** risk factor	**Intermediate** risk factor	**Strong** risk factor	**Very strong** risk factor
Cognitive impairments	◯	◯	◯	◯	◯	◯
Physical disability	◯	◯	◯	◯	◯	◯
Death	◯	◯	◯	◯	◯	◯

SECTION 1: Other health outcomes

1.2 In your opinion, has any '**important"** health state/event been missing from our list?

Note: In this question, "important" is defined as a health state/event for which frailty is at least an **INTERMEDIATE** risk factor.

◯ Yes

◯ No

SECTION 1: Other health outcomes

1.2.1 Please specify the important health states/events which have been missing from our list

1.

2.

3.

4.

5.

6.

7.

8.

9.

10.

SECTION 2: Patient attributes (Specification and rating)

2.1 To what extent do you think each of the following patient attributes influences the progression of frailty and the health outcomes identified in Section 1?

Please note that there are some conditions (e.g. COPD, depression) that have been presented in Section 1. This is because of some evidence indicating a bidirectional relationship between frailty and these conditions, i.e. they can be both a risk factor (influencing the progression of frailty) and a health outcome associated with frailty.

	Does **NOT** influence disease progression	**Very weakly** influence disease progression	**Weakly** influence disease progression	**Intermediately** influence disease progression	**Strongly** influence disease progression	**Very strongly** influence disease progression
Age	○	○	○	○	○	○
Gender	○	○	○	○	○	○
Level of education	○	○	○	○	○	○
Level of income	○	○	○	○	○	○
Smoking status	○	○	○	○	○	○
Marital status (e.g. not married, separated/divorced, widowed)	○	○	○	○	○	○
Traumatic life events (e.g. death of a loved one, serious illness, serious illness loved ones)	○	○	○	○	○	○
Social and community networking (including friends and family as well as broader community networking)	○	○	○	○	○	○
Living environment self-assessment (e.g. not satisfied)	○	○	○	○	○	○
Living area (e.g. living in rural or remote areas)	○	○	○	○	○	○
Polypharmacy	○	○	○	○	○	○
Level of physical activity	○	○	○	○	○	○
Chronic obstructive pulmonary disease (COPD)	○	○	○	○	○	○

	Does **NOT** influence disease progression	**Very weakly** influence disease progression	**Weakly** influence disease progression	**Intermediately** influence disease progression	**Strongly** influence disease progression	**Very strongly** influence disease progression
Heart failure						
Angina						
Myocardial infarction						
Stroke						
Diabetes (with no diabetes-related complications)						
Diabetes (associated with diabetes-related complications)						
Obesity						
Parkinson's Disease						
Impaired vision						
Impaired hearing						
Poor oral health						
Arthritis						
Depression						
Cognitive impairments						
Physical disability						

SECTION 2: Other patient attributes

2.2 In your opinion, has any '**important"** patient attribute been missing from our list?

<u>Note</u>: In this question, "important" is defined as a patient attribute that influences at least **INTERMEDIATELY** the progression of frailty and health outcomes

- ◯ Yes
- ◯ No

SECTION 2: Other patient attributes

2.2.1 Please specify the important patient attributes which have been missing from our list

1.

2.

3.

4.

5.

6.

7.

8.

9.

10.

Additional comments

Please include any further comments about health outcomes and patient attributes

Thank you for completing this survey
