# Supplementary material for: Structuring a conceptual model for cost-effectiveness analysis of frailty interventions
Source: PLoS One. 2019 Sep 11;14(9):e0222049. doi: 10.1371/journal.pone.0222049 (PMC6738928; doi:10.1371/journal.pone.0222049)
Supplement: S1 Table — (PDF) [file pone.0222049.s003.pdf]

Table 1: Health states/event

Findings of the literature review	Rounds 1 and 2 results: Level of agreement (conceptual model inclusion/exclusion)	Round 3 results	Final list of health states/events included in the conceptual model
Hip fracture	Acceptable level of agreement **(Include)**	-	✓
Vertebral fracture	Unacceptable level of agreement (Exclude)	-	X
Other fractures (excluding hip and vertebral fractures)	Moderate level of agreement (Round 3)	Exclude	X
Falls	Acceptable level of agreement **(Include)**	-	✓
Delirium	Moderate level of agreement (Round 3)	**Include**	✓
Incontinence	Moderate level of agreement (Round 3)	Exclude	X
Pressure ulcers	Unacceptable level of agreement (Exclude)	-	X
Sleep disorders	Unacceptable level of agreement (Exclude)	-	X
Residential care admission	Acceptable level of agreement **(Include)**	-	✓
Hospital admission	Acceptable level of agreement **(Include)**	-	✓
Polypharmacy	Moderate level of agreement (Round 3)	Exclude	X
Heart failure	Unacceptable level of agreement (Exclude)	-	X
Angina	Unacceptable level of agreement (Exclude)	-	X
Myocardial infarction	Unacceptable level of agreement (Exclude)	-	X
Stroke	Unacceptable level of agreement (Exclude)	-	X
Diabetes	Unacceptable level of agreement (Exclude)	-	X
Obesity	Unacceptable level of agreement (Exclude)	-	X
Parkinson's disease	Unacceptable level of agreement (Exclude)	-	X
Impaired vision	Unacceptable level of agreement (Exclude)	-	X
Impaired hearing	Unacceptable level of agreement (Exclude)	-	X
Chronic Obstructive Pulmonary Disease (COPD)	Unacceptable level of agreement (Exclude)	-	X
Poor oral health	Unacceptable level of agreement (Exclude)	-	X
Peripheral artery disease	Unacceptable level of agreement (Exclude)	-	X
Renal failure	Unacceptable level of agreement (Exclude)	-	X
Depression	Unacceptable level of agreement (Exclude)	-	X
Cognitive impairment	Moderate level of agreement (Round 3)	Exclude	X
Physical disability	Acceptable level of agreement **(Include)**	-	✓
Death	Acceptable level of agreement **(Include)**	-	✓
