# Supplementary material for: Structuring a conceptual model for cost-effectiveness analysis of frailty interventions
Source: PLoS One. 2019 Sep 11;14(9):e0222049. doi: 10.1371/journal.pone.0222049 (PMC6738928; doi:10.1371/journal.pone.0222049)
Supplement: S2 Table — (PDF) [file pone.0222049.s004.pdf]

Table 2: Patient attributes

Findings of the literature review	Rounds 1 and 2 results: Level of agreement (conceptual model inclusion/exclusion)	Round 3 results	Final list of health states/events included in the conceptual model
Age	Acceptable level of agreement **(Include)**		✓
Gender	Acceptable level of agreement **(Include)**		✓
Level of education	Acceptable level of agreement **(Include)**		✓
Frailty status	Acceptable level of agreement **(Include)**		✓
Level of income	Unacceptable level of agreement (Exclude)		**X**
Smoking status	Moderate level of agreement (Round 3)	Exclude	**X**
Marital status	Unacceptable level of agreement (Exclude)		**X**
Traumatic life events	Unacceptable level of agreement (Exclude)		**X**
Social and community networking	Unacceptable level of agreement (Exclude)		**X**
Living area	Moderate level of agreement (Round 3)	Exclude	**X**
Previous hospital admissions	Acceptable level of agreement **(Include)**		✓
Previous fractures	Acceptable level of agreement **(Include)**		✓
Polypharmacy	Acceptable level of agreement **(Include)**		✓
Level of physical activity	Acceptable level of agreement **(Include)**		✓
Chronic Obstructive Pulmonary Disease (COPD)	Unacceptable level of agreement (Exclude)		**X**
Heart failure	Unacceptable level of agreement (Exclude)		**X**
Myocardial infarction	Unacceptable level of agreement (Exclude)		**X**
Stroke	Acceptable level of agreement **(Include)**		✓
Diabetes (with complications)	Acceptable level of agreement **(Include)**		✓
Obesity	Unacceptable level of agreement (Exclude)		**X**
Parkinson's Disease	Unacceptable level of agreement (Exclude)		**X**
Impaired vision	Unacceptable level of agreement (Exclude)		**X**
Impaired hearing	Unacceptable level of agreement (Exclude)		**X**
Poor oral health	Unacceptable level of agreement (Exclude)		**X**
Arthritis	Unacceptable level of agreement (Exclude)		**X**
Depression	Moderate level of agreement (Round 3)	**Include**	✓
Cognitive impairment	Moderate level of agreement (Round 3)	Exclude	**X**
